# Periostin is identified as a putative metastatic marker in breast cancer-derived exosomes

**DOI:** 10.18632/oncotarget.11663

**Published:** 2016-08-29

**Authors:** Ioulia Vardaki, Sophia Ceder, Dorothea Rutishauser, George Baltatzis, Theodoros Foukakis, Theocharis Panaretakis

**Affiliations:** ^1^ Department of Oncology-Pathology, Cancer Centrum Karolinska, Karolinska Institutet and University Hospital, Stockholm, Sweden; ^2^ Department of Medical Biochemistry and Biophysics, Karolinska Institutet and University Hospital, Stockholm, Sweden; ^3^ Science for Life Laboratory, Stockholm, Sweden; ^4^ Department of Medicine, School of Health Sciences, University of Athens, Athens, Greece

**Keywords:** periostin, biomarkers, exosomes, metastatic, non-metastatic

## Abstract

Breast cancer (BrCa) is the most frequent cancer type in women and a leading cause of cancer related deaths in the world. Despite the decrease in mortality due to better diagnostics and palliative care, there is a lack of prognostic markers of metastasis. Recently, the exploitation of liquid biopsies and in particular of the extracellular vesicles has shown promise in the identification of such prognostic markers. In this study we compared the proteomic content of exosomes derived from metastatic and non-metastatic human (MCF7 and MDA-MB-231) and mouse (67NR and 4T1) cell lines. We found significant differences not only in the amount of secreted exosomes but most importantly in the protein content of exosomes secreted from metastatic versus non-metastatic ones. We identified periostin as a protein that is enriched in exosomes secreted by metastatic cells and validated its presence in a pilot cohort of breast cancer patient samples with localized disease or lymph node (LN) metastasis.

## INTRODUCTION

Breast cancer is divided in four main clinical subtypes based on their molecular characteristics (luminal A, luminal B, human epidermal growth factor receptor 2 (HER2) enriched and basal like) [1, 2]. The detection of the disease in early pre-metastatic stages is very important for evaluating the progression of the disease, the therapeutic strategy and its efficacy. For the last decades there is a decrease in breast cancer mortality, which is mainly attributed to earlier detection and advancements in adjuvant therapy [3]. However, breast cancer remains a leading cancer-related death cause in women worldwide. Current prognostic factors in breast cancer include tumor characteristics such as tumor size, the status of axillary lymph nodes and grade, as well as immunohistochemistry-based tumor markers such as the estrogen receptor (ER), progesterone receptor (PgR), the proliferation marker Ki67 and ERBB2 expression [2, 4–9].

Exosomes are one type of actively secreted extracellular vesicles (EV). They are endosome-derived and their biogenesis and secretion are regulated mainly by ESCRT-dependent and sphingomyelin-dependent processes [10, 11]. Exosomes have been shown to play an important role in cell to cell communication as they contain a wide variety of active proteins, DNA, coding and non-coding RNAs [12–14]. In addition, exosomes secreted from tumor cells can induce chemo-resistance, influence metastatic and invasive potential as well as, tumor growth of recipient cells [15–20].

In the past few years there is an increased interest in EV and exosomes in particular, as a source of biomarkers for a variety of diseases including cancer [21, 22]. It was recently shown that Glypican-1 that is found in circulating exosomes but not in circulating tumor cells, is a biomarker of absolute sensitivity and specificity for pancreatic cancer [23].

In the breast cancer setting, several studies have been performed on profiling exosomes for breast cancer diagnostic markers [24]. Several proteomic studies have been performed on exosomes secreted from human breast cancer cell lines [16, 25]. They have found that exosomes from the highly metastatic cell line MDA-MB-231 cell have a distinct proteome compared to non-metastatic MCF7 cell line; MDA-MB-231 exosomes are enriched in extracellular matrix proteins and proteins related to metastasis and invasion.

Periostin is a secreted protein involved in the adhesion of osteoblasts and it is upregulated in several cancer types (ovarian, non-small cell lung cancer and breast cancer) [26–28]. Periostin is a major component of ECM which is secreted from fibroblasts, highlighting its role in tumor microenvironment regulation [29]. Moreover, periostin is secreted from cancer cells and has the ability to promote their migration and enhance their invasiveness by interacting with integrins [30]. Importantly, this protein has been shown to correlate with increased tumor progression and worse long-term survival outcomes in specific subgroups of breast cancer patients [28, 31].

In this study, we compared the molecular profile of exosomes derived from mouse and human, metastatic cell lines to non-metastatic ones. We found several differentially enriched proteins, many of which were identified in the human breast cancer cell derived exosomes. Notably, periostin was enriched in exosomes secreted from metastatic compared to non-metastatic cell lines and in plasma samples from patients with lymph node metastasis.

## RESULTS

### Characterization of exosomes secreted from 67NR and 4T1 cell lines

**Figure 1:**
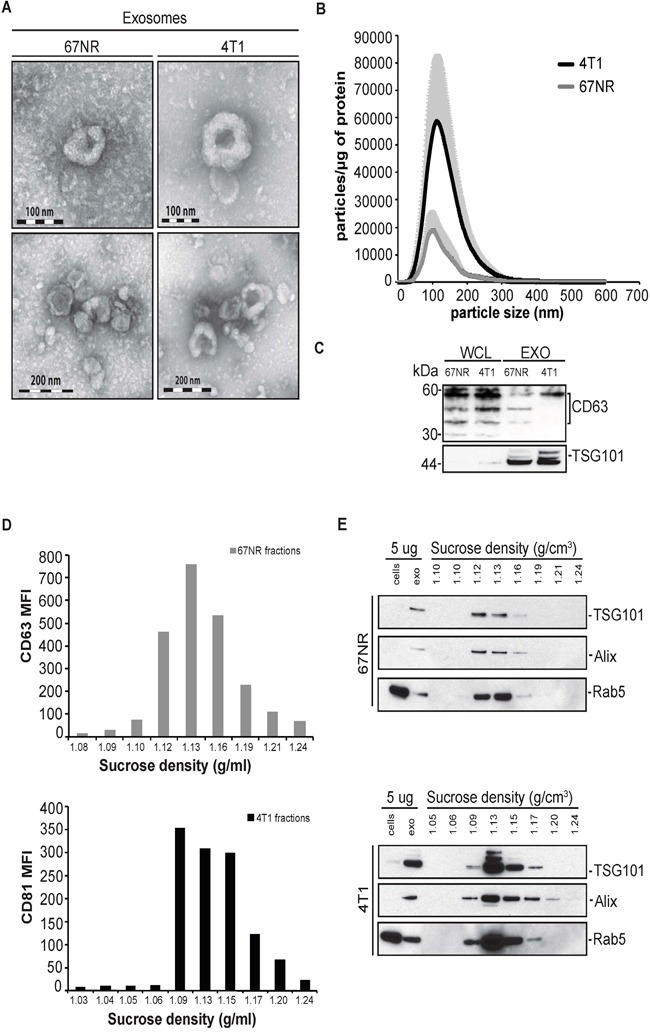
Characterization of extracellular vesicles secreted from non-metastatic mouse breast cancer cell line 67NR and metastatic mouse breast cancer cell line 4T1 **A.** Electron microscopy of 4T1 and 67NR ultracentrifuged pellets revealed the characteristic shape and size of exosomes (*n=1*). **B.** Nanoparticle Tracking Analysis (NTA) of particles secreted from 67NR and 4T1 cell lines. The relative concentration of secreted particles (10^6^ particles/ml), which obtained from Nanosight was normalized with mg of proteins of cell lysates (means +/− SD, *n=3, 5×1 minute movies sequences were measured*). **C.** Western blot analysis of lysates from 67NR and 4T1 cells and exosomes (15μg respectively) probed for a panel of exosomal markers (CD63 and TSG101) *(n=2).* **D.** Flow cytometric analysis of the mean fluorescence analysis of sucrose gradient fractions, for CD81 and CD63 exosomal markers, from 4T1 and 67NR pellets, respectively *(n=2, in triplicates)*. **E.** Western blot analysis of the sucrose gradient fractions from 4T1 and 67NR exosomes (15μg respectively), probed for TSG101, Alix and Rab5 *(n=2)*.

Exosomes were isolated from the supernatants of 67NR and 4T1 cells cultured in exosome depleted medium. Representative images from transmission electron microscopy revealed differences between metastatic and non-metastatic exosomes (Figure 1A). Exosomes from 67NR cells appeared smaller than the 4T1 exosomes, which was confirmed by the Nanoparticle Tracking Analysis (NTA) which revealed mean sizes of 89nm and 102nm, respectively (Figure 1A and 1B). Furthermore, NTA analysis showed that 4T1 cells secrete 3 to 4 times more particles than 67NR cells (Figure 1B). Western blot analysis revealed that the 4T1 exosomes are more enriched in the tetraspanin CD63, whereas there was no difference in the tumor susceptibility gene TSG101 (Figure 1C).

We performed sucrose density gradient on the isolated exosomes from 67NR and 4T1 cells (Figure 1D, 1E). For exosomes isolated from 67NR cells, both flow cytometric immunophenotyping and western blot analysis of the fractions for exosomal markers revealed that the 67NR had a higher concentration between the fractions 1.12-1.16 g/ml whereas the 4T1 exosomes had a broader distribution between the fractions 1.09 to 1.17 (Figure 1D, 1E). In summary, the isolated vesicles from 67NR and 4T1 cell lines have the characteristic morphology, express the characteristic protein markers and have the characteristic physical properties of exosomes.

### Exosomes from 4T1 cells can be uptaken and may influence the migration rate of recipient cells

To examine the functionality of our isolated exosomes we investigated if exosomes from the highly metastatic cell line 4T1 could affect migration of non-metastatic 67NR cells. For that purpose, 67NR or 4T1 cells were plated in conditioned medium and incubated with PKH67-labeled 67NR or 4T1 exosomes for 1, 3 or 6 hours. Syngeneic unlabeled exosomes were used as negative control. The percentage of positive cells indicated that both 67NR and 4T1 exosomes are readily uptaken by 67NR and 4T1 cells (Supplementary Figure 1B and 1D). The highest level of positive cells was observed at 6 hours for both cell lines and there was a tendency for higher uptake of 4T1 than 67NR exosomes from both cell lines. In addition, 4T1 cells seem to uptake, higher amount of both type of exosomes investigated (67NR and 4T1), than 67NR cells (Supplementary Figure 1B and 1D).

Finally in order to investigate the ability of exosomes from the highly metastatic cell line 4T1 to alter the migration rate of non-metastatic cells 67NR we performed a scratch assay (Supplementary Figure 2A and 2C). We found that 4T1 exosomes increased the migration potential of both 67NR and 4T1 cells (Supplementary Figure 2B and 2D).

### Proteomics and bioinformatics analysis of the 67NR and 4T1 exosomes

Proteomic analysis by LC-MS/MS of 67NR and 4T1 cell derived exosomes revealed 440 proteins, of which 130 were present only in the 67NR exosomes, 161 were found only in the 4T1 exosomes and 149 were common between these two types of exosomes (Supplementary Figure 3A and Supplementary Table 1). We found that 94 proteins were identified as unique in 67NR cells and 111 in 4T1 cells. The common proteins between 67NR cells and 67NR exosomes were 15 and the common proteins between 4T1 cells and exosomes were 9 (Supplementary Figure 3A).

We then performed gene ontology analysis using the PANTHER software which revealed that the 4T1 exosomes are primarily enriched with proteins that are involved in cell adhesion and cell-matrix interactions (Supplementary Figure 3B). In the biological processes, there is an increase of exosomal 4T1 proteins that are involved in cell adhesion and cell-matrix interaction such as integrins, laminins and fibronectin. With regard to molecular functions of the exosomal proteins, we found an enrichment of proteins in 4T1 exosomes that have cell surface receptor activity such as extracellular matrix proteins (e.g. integrins), cell adhesion molecules (e.g. tetraspanins) and extracellular matrix linker proteins (e.g. laminins). The strongest evidence for the enrichment of extracellular matrix proteins is found in the analysis of cellular components. In this mode of analysis we found high levels of proteins participating in cell junctions and cell-extracellular matrix interactions (e.g. catenins, matrix metalloproteinases, periostin, laminins and integrins) (Supplementary Figure 3A). In summary, our proteomic analysis, showed that 4T1 exosomes were enriched in proteins related to cell migration and adhesion, functions closely related to cancer metastasis.

### Validation of selected proteins identified by proteomic analysis in 67NR and 4T1 exosomes

**Figure 2:**
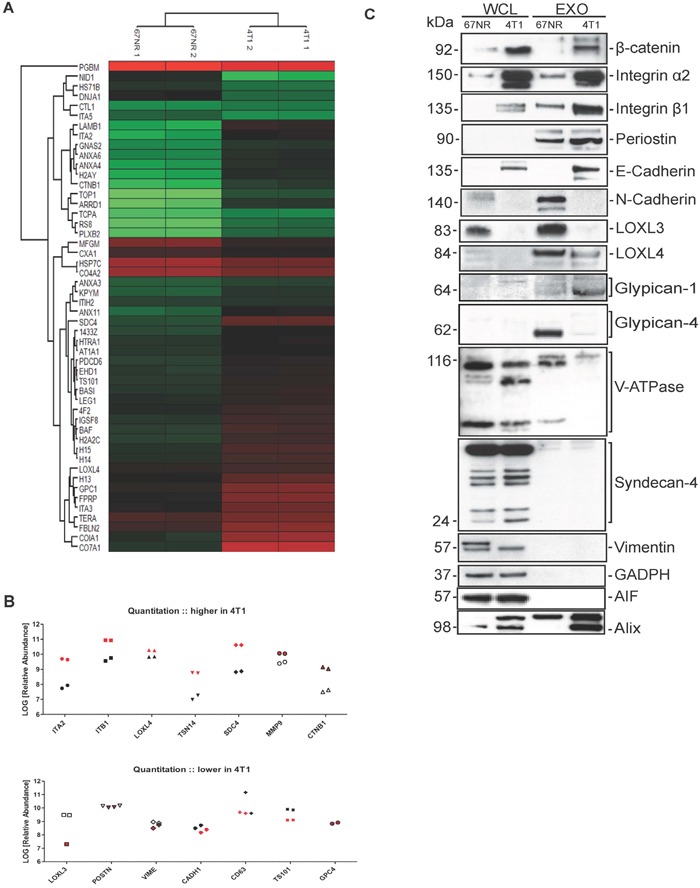
Proteomic analysis of exosomes isolated from 67NR and 4T1 exosomes **A.** Heat map of hierarchical clustering of 50 proteins that were identified with the highest number of peptides, in 67NR and 4T1 exosomes. Red color is corresponding to relatively high abundance of proteins, green relatively low and black color represents equal expression of proteins. **B.** Quantification of proteins that were enriched in 4T1 exosomes (upper graph) and enriched in 67NR but found in low levels in 4T1 exosomes (lower graph). **C.** Validation of proteomics for 67NR and 4T1 exosomes: western blot analysis for 67NR and 4T1 cells and exosomes probed for the indicated proteins. GADPH used as loading control for the cells and AIF as a quality control for the exosome isolation. Equal amount of proteins (10-15μg) was loaded in each Western (*n=2*).

We performed a hierarchical unsupervised clustering of proteins depicted as a heat map of proteins enriched in 67NR and 4T1 exosomes, and found significant differences in protein expression (Figure 2A, top 50 proteins presented). We quantified the abundance levels by using ANOVA pairwise comparisons and selected the proteins that show the biggest difference in abundance levels between the 67NR and 4T1 exosomes (Figure 2B). We then validated by western blotting the top 20 proteins which were revealed by proteomic and gene ontology analysis (Figure 2C). The proteins that were found to be more abundant in 4T1 exosomes in both proteomic analyses are *β*-Catenin and integrin-α2 and integrin-β1 (Figure 2B and 2C). Interestingly, we found an enrichement of periostin in 4T1 compared to 67NR exosomes (Figure 2C). Furthermore, we found that E-cadherin is expressed in both 4T1 cells and exosomes and N-cadherin is mainly expressed in 67NR cells (Figure 2C). Regarding Lysyl oxidase like enzymes, we found that LOXL4 was present in lower levels in 4T1 cells and exosomes than in 67NR cells and exosomes (Figure 2C). LOXL3 could only be detected in 67NR cells and exosomes (Figure 2B and 2C). In this study, the expression of glypican-1 was enriched in 4T1 exosomes and glypican-4 in 67NR exosomes (Figure 2C). The levels of V-ATPase were the same in both 67NR and 4T1 cell lines and in 4T1 exosomes the levels of V-ATPase were lower than in 67NR exosomes (Figure 2C). Finally, we did not detect syndecan-4 and vimentin in neither 67NR nor 4T1 exosomes and the enrichment was found to be equal in 67NR and 4T1 cells (Figure 2C). Programmed Cell death Interacting Protein (Alix) was used as an exosomal marker, as a control of the purity of exosomal isolation we used AIF and GAPDH as a loading control for the whole cell lysates (Figure 2C). Summarizing, we confirmed by western blot analysis, the differential proteomic content of 67NR and 4T1 exosomes, identifying β-catenin, integrin α2 and β1, periostin and E-Cadherin, as putative markers of metastasis in mouse mammary carcinoma cell lines.

### Characterization of exosomes derived from human breast cancer cell lines

**Figure 3:**
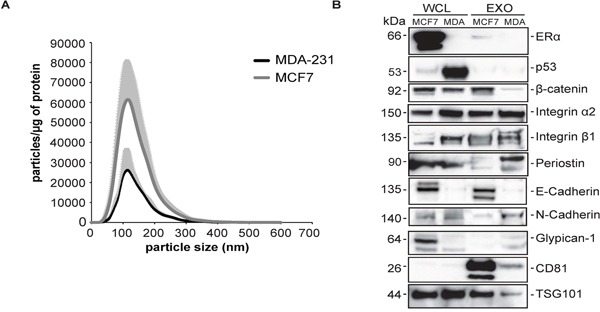
Validation of selected proteins in human breast cancer non-metastatic cell line MCF7 and highly metastatic cell line MDA-MB-231 **A.** Nanoparticle Tracking Analysis (NTA) of particles secreted from MCF7 and MDA-MB-231 cell lines. The relative concentration of secreted particles (10^6^ particles/ml), which obtained from Nanosight was normalized with mg of proteins of cell lysates (means +/− SD, n=3, *5×1 minutes movies sequences were measured*). **B.** Western blot analysis of MCF7 and MDA-MB-231 cells and exosomes, probed for the indicated proteins *(n=2).*

Nanoparticle tracking analysis showed that MCF7 and MDA-MB-231 have the characteristic mean size of exosomes 108 and 106nm respectively with MCF-7 cells secreting more vesicles than the MDA-MB-231 cells (Figure 3A). We examined the presence of ERα in these exosomes and we found ERα in MCF7 cells and exosomes in low levels and, as expected, not in MDA-MB-231 cells and exosomes (Figure 3B). Furthermore, we found the accumulated mutant p53 in MDA-MB-231 cells but not in exosomes (Figure 3B). Periostin and integrin-β1 were enriched in the MDA-MB-231-derived exosomes compared to the MCF7 ones (Figure 3B). β-catenin was expressed in MDA-MB-231 cells but not in their corresponding exosomes, whereas integrin α2 protein levels were similar in the exosomes secreted from metastatic (MDA-MB-231) and non-metastatic cell lines (MCF7) (Figure 3B). Regarding E- and N-Cadherin, we found that MCF7 cells express E-cadherin, whereas MDA-MB-231 cells do not, N-Cadherin levels are higher in MDA-MB-231 exosomes compared to MCF-7 (Figure 3B). CD81 and TSG101 were used as exosomal markers and they were detectable in both exosomes from MCF7 and MDA-MB-231 cell lines. CD81 was highly enriched in exosomes compared to their corresponding exosomes, whereas TSG101 was detectable in the cells and present in their corresponding exosomes (Figure 3B).

### Validation of selected biomarkers in patient samples

**Figure 4:**
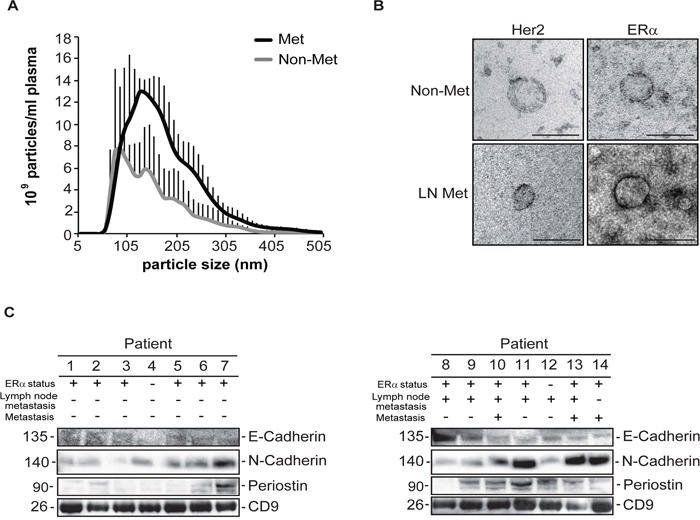
Characterization and validation of exosomes isolated from patients with non-metastatic or metastatic breast cancer **A.** Nanoparticle Tracking Analysis (NTA) of particles isolated from patients' plasma with early or late metastatic breast cancer (means +/− SD, *3 patients with early disease and 3 patients with lymph node metastasis, n=1*). **B.** Immuno-EM of exosomes isolated from patient samples, for the indicated proteins. (Scale bar: 100nm) **C.** Western blot analysis of exosomes (15 μg) secreted from patients with non- or lymph node metastasis, probed for the indicated proteins (n=3).

We isolated exosomes from the plasma of breast cancer patients with localised disease or lymph-node metastasis and/or metastasis to other sites (Figure 4A). We used Nanoparticle Tracking Analysis and we found that exosomes from patients with lymph-node metastasis have a bigger, average size (125 nm) and higher relative concentration (10.9×10^9^ particles/ml of plasma) compared to exosomes from patients with localised breast cancer (95nm and 8.35×10^9^ particles/ml of plasma, respectively) (Figure 4A and Supplementary Figure 4).

We further investigated the cell type of origin in our isolated exosomes from breast cancer patient samples by performing immuno-electron microscopy for Her2 and ERα which are well-known to be expressed in mammary epithelial cells. We found the presence of both of these proteins in a subset of the isolated vesicles (Figure 4B).

We validated our in vitro findings in the extracellular vesicles isolated from the plasma of 14 breast cancer patients, 7 with localized disease and 7 with lymph node (LN) metastasis. We found that mainly periostin and N-Cadherin to a lesser extent were enriched in the extracellular vesicles isolated from the plasma of patients with LN metastasis (Figure 4C). We did not find significant differences for E-cadherin in the extracellular vesicles isolated from patients with LN-metastasis or localized disease (Figure 4C). We used CD9 as a marker for the presence of extracellular vesicles in our preparations (Figure 4C).

## DISCUSSION

In this study, we employed the syngeneic breast cancer model, namely the 67NR and 4T1 mammary epithelial carcinoma cell lines to characterize their exosome molecular profile. We found that 67NR and 4T1 cell-derived exosomes have similar morphology with 4T1 exosomes having a bigger size than the 67NR ones (Figure 1A and 1B). Moreover, the higher exosomal secretion of 4T1 cells compared to 67NR cells, is in accordance with the belief that there is a gradient in exosome secretion from healthy to organ-defined tumor to metastatic cancer [32]. We profiled the molecular protein content and found significant differences. For example, CD63 expression was higher in 4T1 exosomes and had a distinct glycosylation pattern compared to the 67NR ones. CD63 is generally related with less invasiveness in many cancer types, the exception of breast cancer cells in which CD63 has been linked with increased invasiveness and drug resistance, which results from CD63 glycosylation and membrane localization [33, 34].

LC-MS/MS proteomic analysis of 67NR and 4T1 cell-derived exosomes revealed that 4T1 exosomes are enriched in proteins with functions related to migration (adhesion, cell junction and ECM proteins) Based on this analysis, we selected and validated by western blot the enrichement of proteins related to cell adhesion, cell-matrix interactions, integrins and cell-extracellular interactions, such as integrins, cadherins and periostin. Periostin is a secreted protein with a significant role in the adhesion of osteoblasts and has been found to be upregulated in several cancer types including breast cancer [26–28, 35]. Periostin has an important role in breast cancer as it maintains cancer stem cell properties and a potential drug target where periostin's interaction with integrins is disrupted [36, 37]. Interestingly, we found an accumulation of periostin in both 67NR and 4T1 exosomes that could suggest a potential role of these exosomes in the maintenance of cancer stem cell properties (Figure 2C).

We also hypothesized that the exosome-enriched, migration-related proteins may be transferred to recipient cells with low metastatic potential and increase their migration propensity. Indeed we could demonstrate that the 4T1 exosomes could increase the rate of migration of 67NR cells. We also observed that the 4T1 exosomes are more readily uptaken by both 67NR and 4T1 cells. This enhanced uptake could be related to the enrichment of adhesion molecules identified by proteomic analysis in the 4T1 exosomes, leading to enhanced adhesion of these exosomes and subsequent uptake.

An interesting and unexpected finding was that, the amount of exosomes and the enrichment of particular proteins in the exosomes secreted from the human cell lines was opposite to the results obtained in the mouse model. MCF-7 cells, the non-metastatic cell line secreted more particles compared to the MDA-MB-231 cells, as revealed by Nanoparticle Tracking Analysis (Figure 3A). We found the opposite observation in the mouse setting and we are not certain for the reason of this discrepancy. With regard to the molecular content, the expression pattern of integrin-β1 in the mouse and human cell lines is the same, as judged by western blotting, but for integrin-a2 we found no difference in the human setting compared to the strong enrichement identified in the 4T1 exosomes. Another protein that was enriched in metastatic mouse cell-derived exosomes was β-catenin, however we could not recapitulate these findings in the human cell line setting. Regarding E- and N-cadherin, we found that MCF7 express E-cadherin, whereas MDA-MB-231 do not, in alignment with the EMT phenotype. N-cadherin levels are very low in both cell lines, which in agreement with other studies [38, 39]. Interestingly, N-cadherin levels are higher in MDA-MB-231 exosomes compared to MCF-7.

In an attempt to validate our findings in patient samples we used exosomes isolated from the plasma from breast cancer patients with localised disease or lymph-node metastasis, or from patients in which metastatic disease appeared later during follow up (Table 1). Nanoparticle Tracking Analysis, revealed that exosomes from patients with lymph-node metastasis have a bigger, average size and higher relative concentration compared to exosomes from patients with localised breast cancer (Figure 4A). This is in agreement with the findings form the mouse syngeneic cell lines where the highly metastatic cell line 4T1 secreted more particles than the non-metastatic cell line 67NR (Figure 1B). However, it is not in agreement with the data we obtained from our human model cell lines, further highlighting the difference between in vitro and ex vivo findings.

**Table 1:** Patients' characteristics from which exosomes were isolated from their plasma

Patient	Age at Diagnosis	Tumor stage	Nodal Metastasis	Later Metastasis	Grade	ER status	PGR status	HER2	Outcome
**1**	42	T2	−	−	T3	+	+	+	Relapse Free
**2**	52	T2	−	−	T3	+	+	−	Relapse Free
**3**	54	T1	−	−	T2	+	+	−	Relapse Free
**4**	65	T1	−	−	T3	−	−	−	Relapse Free
**5**	49	T2	−	−	T2	+	+	−	Relapse Free
**6**	60	T1	−	−	T2	+	+	+	Relapse Free
**7**	69	T1	−	−	T3	−	−	+	Relapse Free
**8**	64	T2	+	−	T2	+	+	−	Relapse Free
**9**	46	T2	+	−	T1	+	+	−	Relapse Free
**10**	42	T3	+	+	T3	+	+	−	Relapse Free
**11**	56	T1	+	−	T2	+	+	−	Relapse Free
**12**	28	T2	+	−	T1	+	−	−	Relapse Free
**13**	42	T1	+	+	T2	+	+	−	Alive with Disease
**14**	55	T2	−	+	T3	−	−	−	Dead of Disease

Concerning the validation of putative biomarkers in breast cancer patient samples, periostin was detected in higher levels in more samples from exosomes with metastatic breast cancer than in exosomes with non-metastatic breast cancer (Figure 4C). N-cadherin, E-cadherin and ERα were detectable in both categories of patients. These findings were expected since the isolated exosomes originated from all cell types of the patients and for example ERα is expressed physiologically from other cell types, apart from the tumour cells [40].

In summary, periostin was found to be enriched in mouse and human breast cancer exosomes compared to their parental cells and in exosomes from patients with lymph node metastasis compared to exosomes from patients with localized disease. These data suggest that periostin could serve as biomarker for metastatic disease. Additional samples from a bigger cohort of patient samples should be analyzed in order to strengthen the clinical significance of these findings.

## MATERIALS AND METHODS

### Ethical permissions and patient samples

This study was approved by the local ethics committee. A written informed consent was signed by all patients for the collection of plasma samples at the time of breast cancer diagnosis. The patient samples we have are all primary breast cancer half of which were with N+ disease (primary breast cancer with lymph node metastasis at diagnosis) and patients that relapsed (metastasis) during the follow-up.

### Cell culture

Murine mammary tumor cell line 67NR was purchased from Karmanos Cancer Institute, MI, USA and murine mammary tumor cell line 4T1 were provided by Dr. Janine Erler, University of Copenhagen, Denmark. Human mammary tumor cell line MCF7 were provided from Dr. Jonas Berghs, Karolinska Institutet, Sweden and MDA-MB-231 cell line was provided by Dr. Massague, Howard Hughes Medical Institute, USA. All cell lines were cultured at 37°C in 5% CO_2._ Cell lines 67NR, MDA-MB-231 and MCF7 were grown in Dulbecco's modified Eagle's medium (DMEM) (HyClone, Thermo Scientific, Bremen, Germany), 4T1 cell line was grown in RPMI-1640 medium. All were supplemented with 10% heat-inactivated FBS (GIBCO, Invitrogen Life Technologies, Paisley, UK), 2 mM L-glutamine (GIBCO, Invitrogen Life Technologies, Paisley, UK), 100U/ml penicillin with 100μg/ml streptomycin (GIBCO, Invitrogen Life Technologies, Paisley, UK).

### Chemicals and antibodies

For western blot, cells and exosomes were lysed in RIPA buffer adjusted to pH 7.4 (containing 50mM Tris, 150mM NaCl, 1mM EDTA, 1% IGEPAL, 1% glycerol) with PhosSTOP Phosphatase Inhibitor Cocktail (Roche, Basel, Schweiz), Complete Protease Inhibitor Cocktail (Roche, Basel, Schweiz), 100mM vanadate; prepared from Sodium Orthovanadate (Sigma-Aldrich, St. Louis, MO, USA), 1 mM dithiothreitol (DTT) (Sigma-Aldrich, St. Louis, MO, USA). For protein analysis with MS cells and exosomes were lysed in 8M urea and diluted using AmBic. Protein concentration was determined by BCA assay (Pierce, Thermo Scientific, Bremen, Germany).

For western blotting, primary antibodies against the following targets were used: TSG101 (Abcam, ab30871, Abcam Cambridge, MA), Alix (Cell Signalling, #2171, Cell Signalling Technology, Danvers, MA, USA), Rab5 (Cell Signalling #3547, Cell Signalling Technology, Danvers, MA, USA), CD63 (sc-15363, Santa Cruz Biotechnology, Santa Cruz, CA, USA Cruz), b-catenin (Cell Signalling #8480, Cell Signalling Technology, Danvers, MA, USA), E-cadherin (BD 610181, BD BIOSCIENCES, NJ, USA), integrin-b1 (Cell Signalling #4706, Cell Signalling Technology, Danvers, MA, USA), integrin-a2 (Abcam, ab13357, Abcam, Cambridge, UK) LOXL3 and LOXL4 (sc-377216 and sc-66952 respectively, Santa Cruz Biotechnology, Santa Cruz, CA, USA), periostin (sc-67233, Santa Cruz Biotechnology, Santa Cruz, CA, USA), N-cadherin (BD 610920, BD BIOSCIENCES, NJ, USA), tetraspanin-14 (Acris, AP32800PU-N, San Diego, USA), V-ATPase (sc-28801, Santa Cruz Biotechnology, Santa Cruz, CA, USA), vimentin (Cell Signaling #5741, Cell Signalling Technology, Danvers, MA, USA), Glypican-1 (sc-365000, Santa Cruz Biotechnology, Santa Cruz, CA, USA), Glypican-4 (sc-247028, Santa Cruz Biotechnology, Santa Cruz, CA, USA), Syndecan-4 (sc-15350, Santa Cruz Biotechnology, Santa Cruz, CA, USA), ERα (sc-8002, Santa Cruz Biotechnology, Santa Cruz, CA, USA), CD81 (sc-166028, Santa Cruz Biotechnology, Santa Cruz, CA, USA). For secondary antibody HRP-conjugated anti-rabbit (Cell Signalling, #7074, Cell Signalling Technology, Danvers, MA, USA), anti-mouse (Cell Signalling #7076, Cell Signalling Technology, Danvers, MA, USA) or anti-goat (sc-2020, Santa Cruz Biotechnology, Santa Cruz, CA, USA) were incubated and the protein bands were detected by western Lightning ECL or Plus-ECL (PerkinElmer, Santa Clara, CA, USA) and CL-XPosure Film (Thermo Scientific, Bremen, Germany).

PE-Conjugated antibodies (Biolegend, San Diego, CA) used for flow cytometry against 4 μm-sized latex beads (Invitrogen Life Technologies, Paisley, UK, Paisley, UK) were against CD63 and CD81 IgG Isotype Ctrl-PE (BioLegend, San Diego, CA) was used as control for CD81 and IgG2a, κ isotype Ctrl-PE was used as a control for CD63 and CD9.

### Exosome isolation

Exosomes were isolated from cells cultured in conditioned medium, as previously described [14]. Briefly, for the depletion of exosomes from medium, 30% FBS medium was prepared and ultracentrifuged for 16 hours at 100,000g at 4°C. The supernatant was filtered into a vacuum-connected 0.22μm filter and diluted with medium supplemented with all the nutrients and antibiotics, in order to prepare the conditioned medium.

Cells were cultured until they reached ~80% confluency and the supernatants were centrifuged at 100g for 10 minutes at room temperature to remove cell debris and filtered through a 0.22μm filter. Supernatants were frozen at −20°C and thawed at 4°C, then ultracentrifuged for 120 minutes at 120,000g at 4°C. The supernatant was discarded and the pellet was washed in phosphate buffered solution (PBS) and ultracentrifuged for 120 minutes at 120,000g at 4°C. Exosomes used for western blotting were resuspended in 1xRIPA lysis buffer whereas exosomes used for functional studies were resuspended in conditioned medium or PBS.

For exosome isolation from patient plasma samples, whole blood was acquired and centrifuged (within one hour from blood sampling) at 1300g in 20 C for 10 min. The supernatant was centrifuge again at 2400g in 20 C for 15 min. The supernatant was stored in −20 C for max 2 weeks before moving to the −80 C freezer. Subsequently, 2 ml from each sample were used. The samples were gently mixed by two cycles of rotation for 2 minutes, at 4°C. Then, the samples were centrifuged for 20 seconds at 500g. The supernatant was diluted in PBS, until a final volume of 6 ml and centrifuged for 2 hours at 120,000g. From the resulting pellet, 40μl were kept and resuspended in PBS for secretion studies. For western blot, the pellet was lysed with 1xRIPA.

### Sucrose gradient

Sucrose gradient ranging from 0.2 to 2 M sucrose was prepared as previously described [41]. Exosomes were placed on the surface of the sucrose gradient and ultracentrifuged at 120,000g at 4°C, for 20 hours. The fractions were collected and used for exosomal characterization; for western blotting and flow cytometry the fractions were ultracentrifuged at 120,000g for 2 hours, 4°C.

### Protein digestion

10μg of extracted protein from each sample were dissolved in 0.1% ProteaseMax, 50 mM ammonium bicarbonate and 10% acetonitrile. Resulting protein solutions were incubated for 30 minutes at 50°C followed by an additional bath soncication of 10 min at room temperature. Samples were centrifuged and directly subjected to a tryptic digestion protocol carried out by a liquid handling robot (MultiProbe II, Perkin Elmer). This included protein reduction in 5 mM DTT at 56°C and alkylation in 15 mM iodacetamide for 30 min at room temperature in the dark. Trypsin was added in an enzyme to protein ratio of 1:30 and digestion was carried out over night at 37°C.

### Liquid chromatography tandem mass spectrometry

For each type of exosomes, 67NR and 4T1 two technical duplicates of pooled samples were used. Tryptic peptides were cleaned with C18 StageTips (Thermo Fisher Scientific Inc) and the resulting peptide mixture was injected into a nano-Ultimate system (Thermo Scientific, Bremen, Germany) in-line coupled to a QExactive mass spectrometer (Thermo Scientific, Bremen, Germany). The chromatographic separation of the peptides was achieved using an in-house packed column (C18-AQ ReproSil-Pur^®^, Dr. Maisch GmbH, Germany) with the following gradient: 5−35% acetonitrile in 89 minutes, 48−80% ACN for 5 minutes and 80% ACN for 8 minutes all at a flow rate of 300 nl/min.

The MS acquisition method was comprised of one survey full scan ranging from m/z 300 to m/z 1650 acquired with a resolution of R= 70,000 at m/z 400, followed by data-dependent HCD scans from maximum ten most intense precursor ions with a charge state ≥ 2. MS2 scans were acquired with a resolution of R=17,500, a target value of 2e5, isolation width was set to 4 and normalized collision energy to 26.

### Data analysis

Tandem mass spectra were extracted using Raw2MGF (in-house developed software), and the resulting mascot generic files were searched against a SwissProt protein database (reversed protein sequences had been added to database for decoy search) using the Mascot 2.3.0 (Matrix Science Ltd.). Mascot was set up to search a concatenated SwissProt protein database (selected for *Mus musculus*) using trypsin and allowing for one missed cleavage sites. Peptide mass tolerance was set to 10 ppm and 0.02 Da for the fragment ions. Carbamidomethylation of cysteine was specified as a fixed modification, whereas oxidation of methionine and deamidation of asparagine and glutamine were defined as variable modifications.

Quantitative information was extracted using in-house developed software Quanti. This software performs extracted ion current quantification. For quantitative purposes only peptides identified with a Mascot Score higher than 18 were selected. Such a threshold was set to fulfill condition of no more than 1% of FDR over total peptide population. Only proteins with at least two peptides were considered for quantitation. The hierarchical clustering in Figure 2A was generated in Perseus (MaxQuant 2.4) and was done for row and column tree using the following parameters: euclidean distance, linkage method average and k-means preprocessing. The input data were the most abundant 50 proteins.

### Flow cytometry

To detect surface exosomal markers in exosomes by flow cytometry, firstly exosomes were bound to latex beads (Invitrogen, Life Technologies, Paisley, UK, A37304) and then stained with conjugated fluorescent antibodies. Briefly, 20μl of 4μm latex beads were washed with 1ml PBS twice and incubated with 5μg of purified exosomes or 100μl of each sucrose fraction for 30 minutes with gentle agitation, at room temperature. Next PBS was added to a final volume of 1ml, and incubated on a test tube rotator wheel overnight at 4°C. Then the beads were washed and resuspended in BCB blocking buffer and incubated for 30 minutes with gentle agitation at room temperature. The beads were then washed again, divided into several tubes and fluorescent conjugated antibodies diluted 1:50 in PBS, against CD63, and CD81 were added. The beads were incubated for 30 minutes, washed twice and ran on a FACS Calibur flow cytometer (BD biosciences, NJ, USA). The data were analyzed and quantified by using the softwares, CellQuest (BD BIOSCIENCES) and FlowJo (Treestar).

### Electron microscopy

For exosome immunogold labeling for electron microscopy, exosomes were prepared and labeled according to the protocol of Thery et al [41]. In brief, isolated exosomes were resuspended in 2% paraformaldehyde (PFA), adsorbed unto nickel Formvar-carbon coated electron microscopy grids (200 mesh), blocked with PBS/5% (w/v) BSA and incubated with antibodies for Her-2 (MSK044, Zytomed Systems, Berlin, Germany), ERα (sc-8002, Santa Cruz Biotechnology, Santa Cruz, CA, USA) and Glypican-1 (sc-101827, Santa Cruz Biotechnology, Santa Cruz, CA, USA) for 30 min. Subsequently grids were washed, blocked and incubated with Donkey Anti-Mouse IgG H&L 10nm Gold antibody (Abcam, Cambridge, United Kingdom). Grids were washed, fixed with 1% glutaraldehyde, contrasted with 4% uranyl acetate and finally embedded in a mixture of 4% uranyl acetate and 2% methyl cellulose.

Grids were visualized on a Morgagni 268 Electron Microscope (FEI, Eindhoven, The Netherlands) and photographed with the Morada Soft Imaging System (Olympus Corporation, Hamburg, Germany).

### Western blotting

Cells and exosome pellets were lysed using 1xRIPA buffer (containing 50mM Tris, 150mM NaCl, 1mM EDTA, 1% IGEPAL, 1% glycerol), supplemented with Complete Protease Inhibitor Cocktail (Roche, Basel, Schweiz), PhosSTOP Phosphatase Inhibitor Cocktail (Roche, Basel, Schweiz), 100mM vanadate (Invitrogen Life Technologies, Paisley, UK), 1mM dithiothreitol (DTT) (Sigma Aldrich, St. Louis, MO, USA). Exosomes in PBS suspension were lysed using 4xRIPA but to a final concentration of 1XRIPA. Protein concentration was determined with Bradford assay using Bradford solution (Bio-rad). Equal amount of proteins (10-15μg) for each Western Blot, were boiled at 90°C for 5 min with LDS (Invitrogen Life Technologies, Paisley, UK) and DTT. Proteins were separated in 10 or 12% Bis-Tris gels (Invitrogen Life Technologies, Paisley, UK) in 1xMES running buffer (Invitrogen Life Technologies, Paisley, UK) containing anti-oxidant (Invitrogen Life Technologies, Paisley, UK), according to manufacturer's instructions. Proteins were transferred to PVDF membranes (PerkinElmer, Santa Clara, CA, USA), in transfer buffer (Invitrogen Life Technologies, Paisley, UK) containing 20% methanol. Membranes were blocked in 5% (w/v) non-fat milk in 1xTBS containing 0.1% Tween-20 for 1 hour in room temperature and then incubated with primary antibodies in the appropriate dilutions (1/500-1/1000) and incubated overnight at 4°C. HRP-conjugated antibodies in 1/2000 dilution were used as secondary antibodies and the membranes were incubated for 1 hour at room temperature. Washing steps between incubations with antibodies were performed with 1xTBS containing 0.1% Tween-20. ECL or ECL+ (PerkinElmer, Santa Clara, CA, USA) were used for the developing with X-ray film (CL-exposure Film, Thermo Scientific, Bremen, Germany).

### Nanoparticle tracking analysis (NTA)

For the secretion study, NS500 (NanoSight Limited, London, UK) was used, equipped with an 8 mega pixel camera (Andor Technology, Tokyo, Japan) and a 405nm laser, in order to measure the size and determine concentration of exosomes. NTA v2.3.0.17 software (NanoSight Limited) was used for both data acquisition and analysis. Three samples from each exosome type were measured and their average was calculated. The duration of each video was 5×1 minutes. During the analysis procedure the camera level was 14 and the detection threshold was 7.

For the patient samples analysis NTA v3.0 software was used. Three samples from node negative and four from node positive patients were used and their average was calculated. The camera level was set at 8 and the detection threshold was 7.

### Exosome PKH67 labeling

4T1 exosomes were labelled with PKH67 green fluorescent (PKH67 Green Fluorescent Cell Linker Midi Kit for General Cell Membrane Labelling, #MIDI67, Sigma-Aldrich, St. Louis, MO, USA) according to the manufacturer's instructions. Briefly, exosome pellets were labelled with 2.5 μM of PKH67 dye in 400 μl of diluent C for 5 minutes. Afterwards, blocking buffer (1% of bovine serum albumin) was added and incubated for 1 minute. Finally, exosomes were washed with PBS by ultracentrifugation at 120,000g, for 2 hours, 4°C. PKH67-labeled exosomes were then resuspended in 150μl PBS.

### Exosome uptake assay

In 48 well plates, 8×10^4^ or 5×10^4^ cells/well were seeded from 67NR and 4T1 cells respectively, in conditioned medium, in a final volume of 400μl. After cells reached ~80% confluency 1.2μg/ml 67NR or 4T1 PKH67 labeled exosomes were added, in final volume 400μl and incubated for 1, 3 or 6 hours and cells were harvested, washed 3 times with 0,5% BSA in PBS and the percentage of green fluorescence intensity (of 10,000 gated events in the FSC/SSC dot plot) was measured by using the BD FACS LSRII flow cytometer (n=2 in triplicates). The results were analyzed by using the BD FACSDiva software (BD BIOSCIENCES, NJ, USA).

### Exosome uptake assay for fluorescent microscopy

In 24-well plate glass coverslips were placed and 2×10^4^ cells/well were seeded from 67NR or 4T1 cells, in conditioned medium, in final volume of 500μl. After 16 hours in 67NR cells 0.3μg/ml of PKH67 labeled exosomes were added, whereas in 4T1 cells, after 40 hours 0.48 μg/ml were added and incubated for 3 hours (the exosomes' concentration was adjusted according to ratio cells/exosomes in order to be consistent with the uptake experiment which was previously described). The images were captured on a DAS Leitz DM RB microscope with a Hamamatsu C4880 dual-mode cooled CCD camera and representative images were processed using Photoshop software (Adobe).

### Scratch assay

In a 24 well plate, 7.5×10^4^ 67NR or 4T1 cells were seeded in conditioned media, in final volume of 500μl. After 16 hours, they were scratched with a 200μl pipette tip and 3μg/ml of 67NR or 4T1 exosomes were added. Photos from the scratched areas were taken in 0, 24, 48 hours for 67NR cells and in 0, 12, 24 hours for 4T1 cells, using a phase contrast microscope. Multiple digital images of the wound were captured at the indicated time points (0h, 12, 24h) after inducing the wound and the closure of the wound was calculated by using the distance measurement capabilities in the image-j software (NIH image, United States).

## SUPPLEMENTARY FIGURES AND TABLE




