# Supplementary material for: Periostin is identified as a putative metastatic marker in breast cancer-derived exosomes
Source: Oncotarget. 2016 Aug 29;7(46):74966–78. doi: 10.18632/oncotarget.11663 (PMC5342715; doi:10.18632/oncotarget.11663)
Supplement: Supplementary file 1 [file oncotarget-07-74966-s001.pdf]

Periostin is identified as a putative metastatic marker in breast cancer-derived exosomes

SUPPLEMENTARY FIGURES AND TABLE

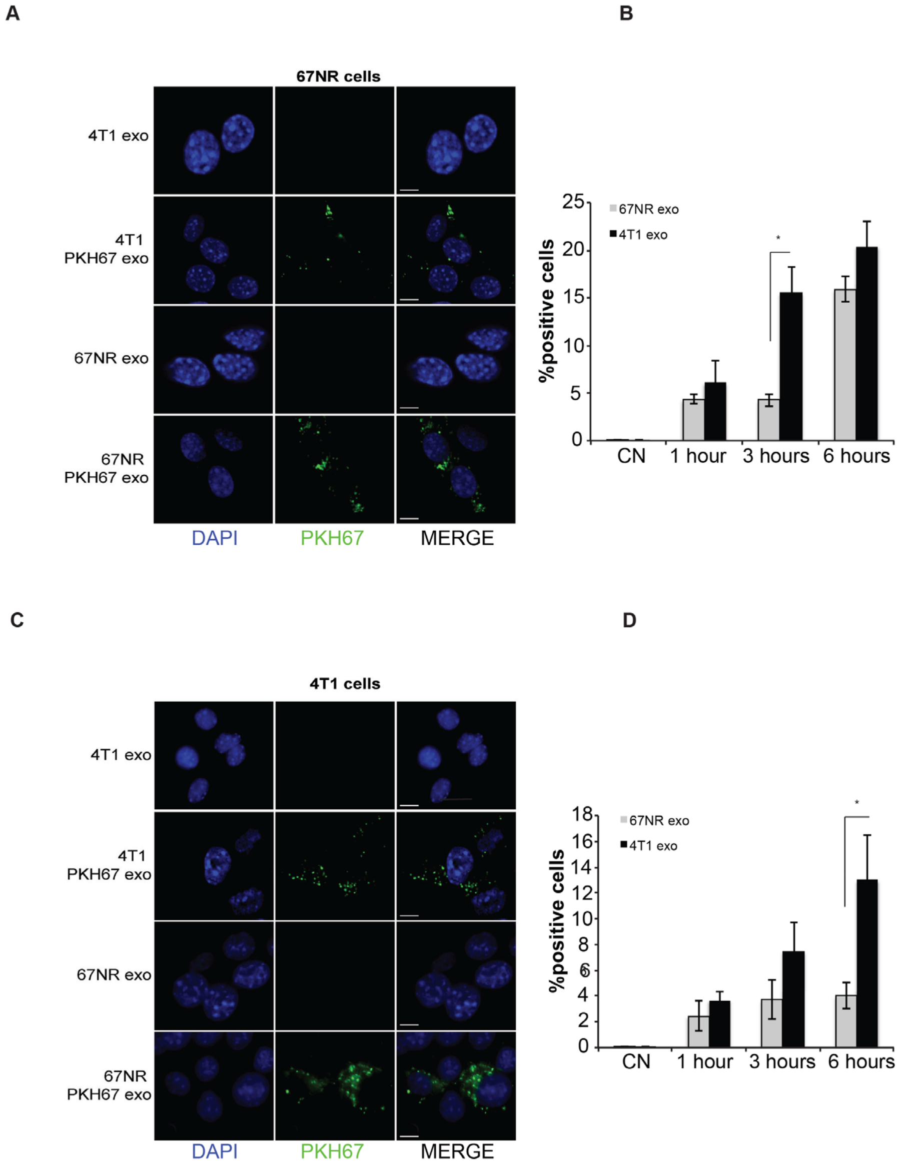

Supplementary Figure S1: 4T1 cell-derived exosomes are uptaken more efficiently than 67NR cell-derived exosomes. **A, C.** Representative confocal images of 67NR and 4T1 cells respectively, incubated for 3 hours with PKH67-labeled exosomes *(n=2)*. **B, D.** Percentage of PKH67-positive cells, after 1,3 or 6 hours incubation with PKH67 labeled 67NR or 4T1 exosomes with B. 67NR cells or D. 4T1 cells. (means +/- SD) *p <0.05, **p<0.01, p***<0.001*(n=2, in triplicates)*.

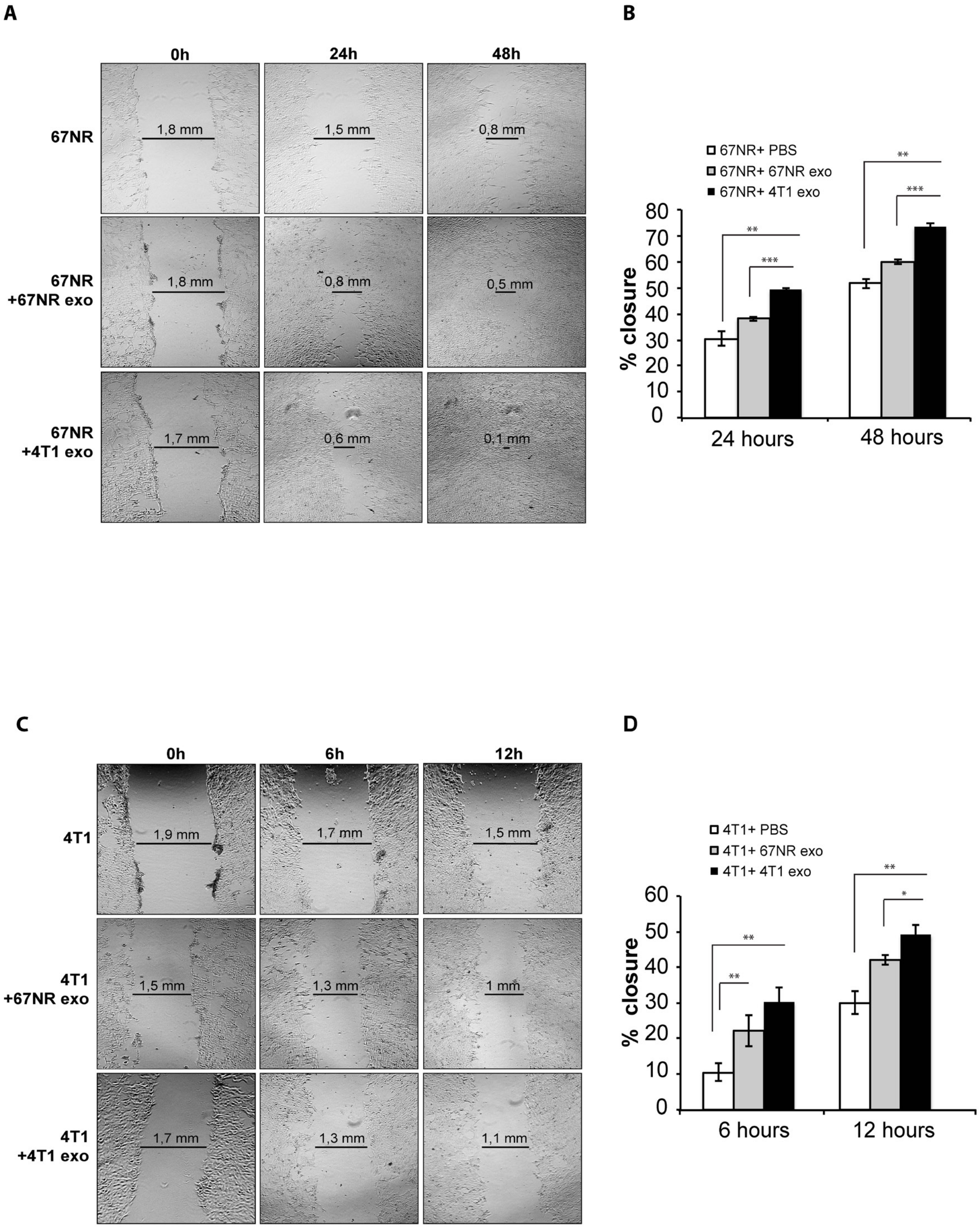

Supplementary Figure S2: Exosomes from 4T1 cells affect the migration rate of recipient cells. Scratch assay revealed that exosomes from 4T1 cells increased the motility of 67NR and 4T1 cells more than 67NR exosomes. **A.** Representative images of scratch assay on 67NR cells at 0, 24 and 48 hours of incubation. **C.** Representative images of scratch assay on 4T1 cells at 0, 12 and 24 hours of incubation. **B, D.** Graphs, which represent the percentage of closure of 67NR and 4T1 cells, respectively. (means +/- SD) *p <0.05, **p<0.01, p***<0.001 *(n=2, in triplicates).*

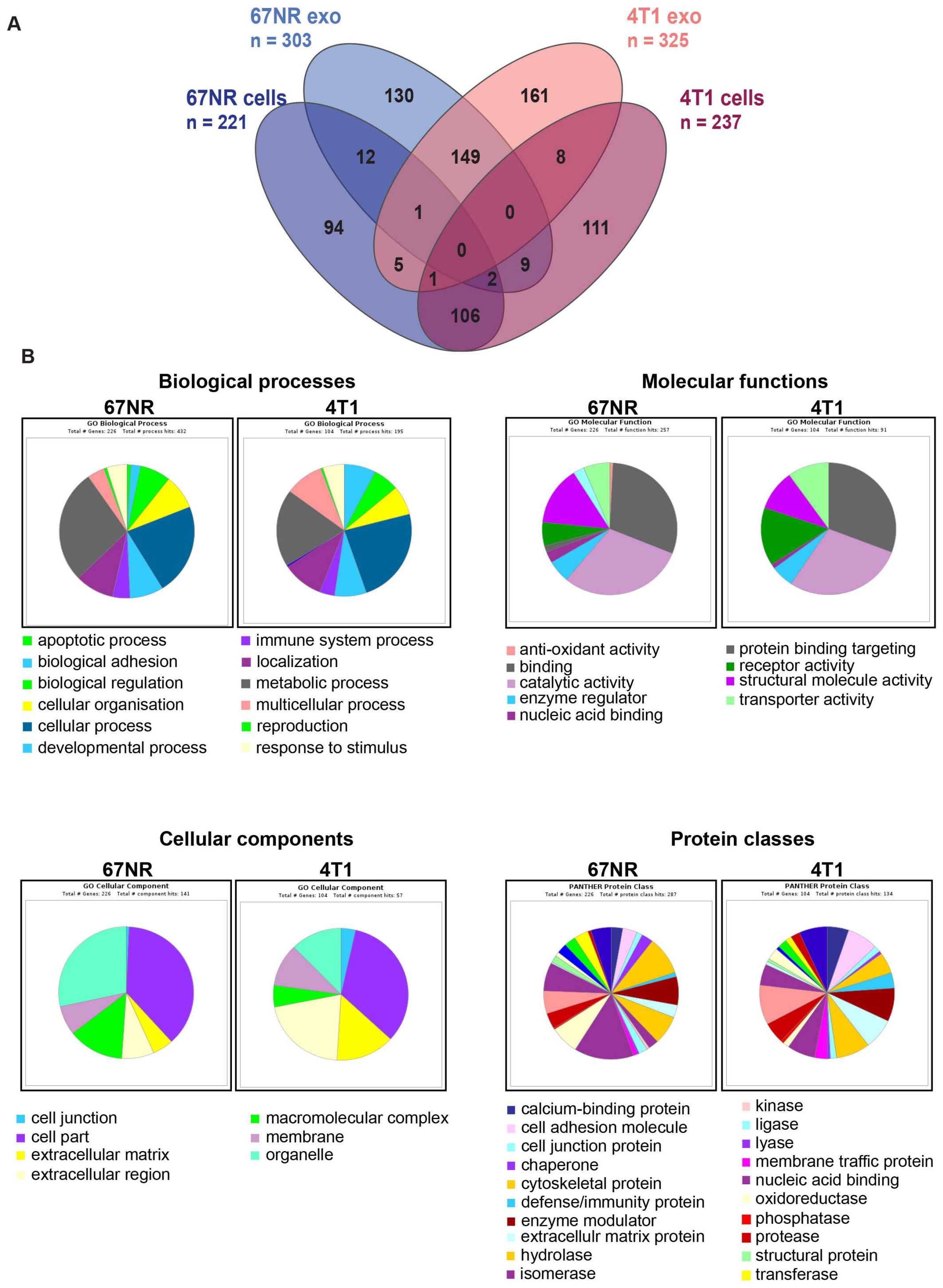

Supplementary Figure S3: Bioinformatics analysis of 67NR and 4T1 exosome proteome. A. Venn Diagram created from the unique and common proteins identified in 67NR and 4T1 cells and exosomes. **B.** Pie charts generated using the classification analysis software PANTHER 9.0 regarding biological processes, molecular functions, cellular components and protein classes.

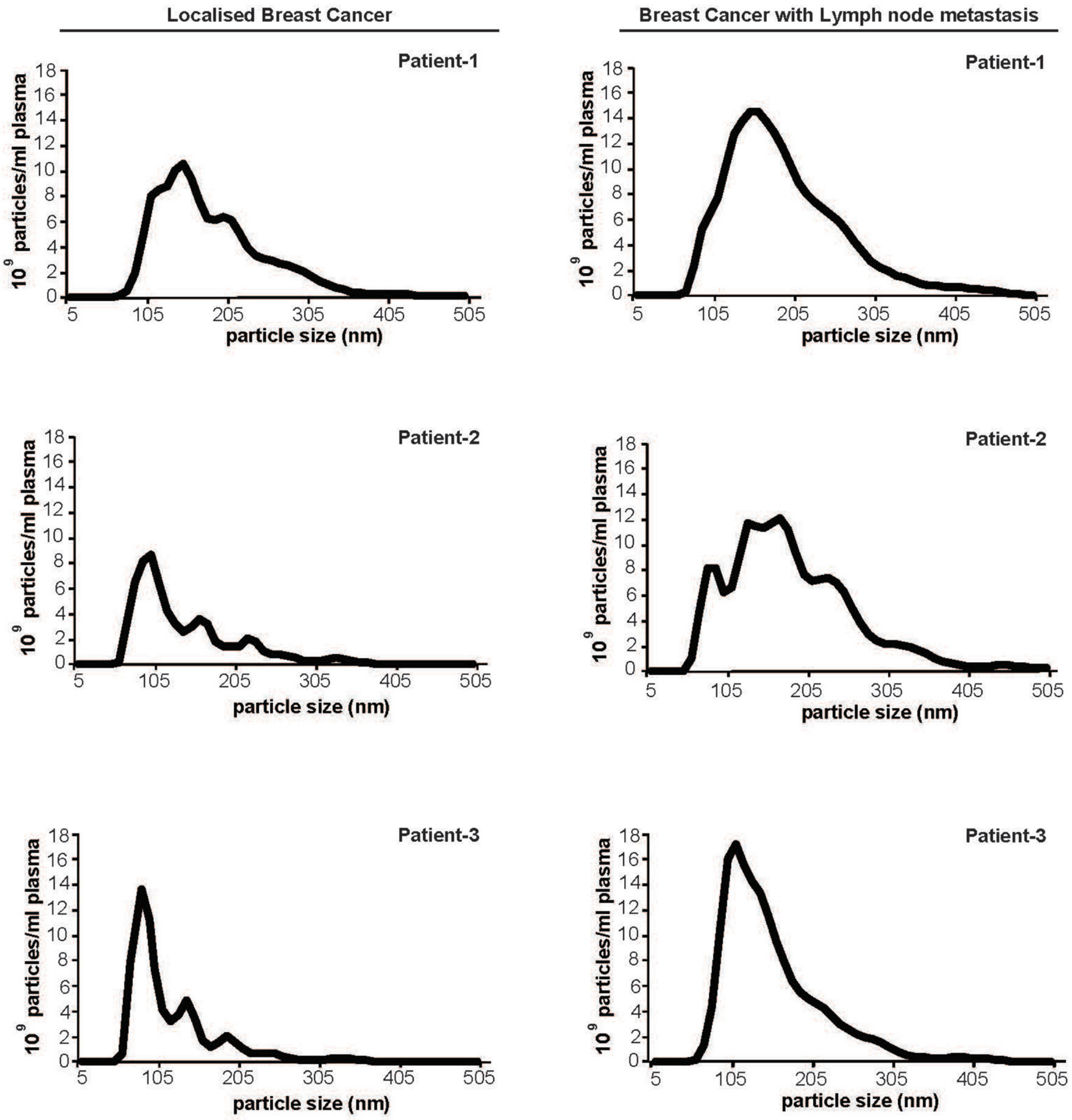

Supplementary Figure S4: Nanoparticle tracking analysis of 3 individual breast cancer patients with localized disease and 3 patients with breast cancer patients with lymph node metastasis (representative curves of the 5x1 minute videos captured).

Supplementary Table S1: Identified proteins from the comparative proteomics analysis of exosomes secreted from the 67NR and 4T1 cells.

See Supplementary File 1
